# Performance of the Hypotension Prediction Index with non-invasive arterial pressure waveforms in non-cardiac surgical patients

**DOI:** 10.1007/s10877-020-00463-5

**Published:** 2020-01-27

**Authors:** Kamal Maheshwari, Sai Buddi, Zhongping Jian, Jos Settels, Tetsuya Shimada, Barak Cohen, Daniel I. Sessler, Feras Hatib

**Affiliations:** 1 Department of General Anesthesiology, Anesthesiology Institute, Cleveland Clinic, Cleveland, OH USA; 2 Edwards Lifesciences Critical Care, Irvine, CA USA; 3 Department of Outcomes Research, Anesthesiology Institute, Cleveland Clinic, Cleveland, OH USA; 4 Department of Anesthesiology, National Defense Medical College, Tokorozawa, Saitama Japan; 5 Division of Anesthesia, Critical Care, and Pain Medicine, Tel-Aviv Medical Center, Sackler Faculty of Medicine, Tel-Aviv University, Tel Aviv, Israel

**Keywords:** Non-invasive blood pressure, Hypotension prediction, Intraoperative hypotension, Machine learning, Hypotension Prediction Index

## Abstract

An algorithm derived from machine learning uses the arterial waveform to predict intraoperative hypotension some minutes before episodes, possibly giving clinician’s time to intervene and prevent hypotension. Whether the Hypotension Prediction Index works well with noninvasive arterial pressure waveforms remains unknown. We therefore evaluated sensitivity, specificity, and positive predictive value of the Index based on non-invasive arterial waveform estimates. We used continuous hemodynamic data measured from ClearSight (formerly Nexfin) noninvasive finger blood pressure monitors in surgical patients. We re-evaluated data from a trial that included 320 adults ≥ 45 years old designated ASA physical status 3 or 4 who had moderate-to-high-risk non-cardiac surgery with general anesthesia. We calculated sensitivity and specificity for predicting hypotension, defined as mean arterial pressure ≤ 65 mmHg for at least 1 min, and characterized the relationship with receiver operating characteristics curves. We also evaluated the number of hypotensive events at various ranges of the Hypotension Prediction Index. And finally, we calculated the positive predictive value for hypotension episodes when the Prediction Index threshold was 85. The algorithm predicted hypotension 5 min in advance, with a sensitivity of 0.86 [95% confidence interval 0.82, 0.89] and specificity 0.86 [0.82, 0.89]. At 10 min, the sensitivity was 0.83 [0.79, 0.86] and the specificity was 0.83 [0.79, 0.86]. And at 15 min, the sensitivity was 0.75 [0.71, 0.80] and the specificity was 0.75 [0.71, 0.80]. The positive predictive value of the algorithm prediction at an Index threshold of 85 was 0.83 [0.79, 0.87]. A Hypotension Prediction Index of 80–89 provided a median of 6.0 [95% confidence interval 5.3, 6.7] minutes warning before mean arterial pressure decreased to < 65 mmHg. The Hypotension Prediction Index, which was developed and validated with invasive arterial waveforms, predicts intraoperative hypotension reasonably well from non-invasive estimates of the arterial waveform. Hypotension prediction, along with appropriate management, can potentially reduce intraoperative hypotension. Being able to use the non-invasive pressure waveform will widen the range of patients who might benefit.

**Clinical Trial Number**: ClinicalTrials.gov NCT02872896.

**Electronic supplementary material:**

The online version of this article (10.1007/s10877-020-00463-5) contains supplementary material, which is available to authorized users.

## Introduction

The association between hypotension and serious complications and mortality in non-cardiac surgical patients is well established [1–7]. There is also limited evidence from a randomized trial that the relationship is causal [8]. Timely and appropriate treatment of hypotension reduces overall hypotension exposure. Even better would be to predict hypotension, thus allowing the clinicians time to intervene and potentially moderate or even prevent hypotension.

Recently, an algorithm (Hypotension Prediction Index, HPI) based on machine learning was developed which predicts intraoperative hypotension (defined as mean arterial pressure < 65 mmHg sustained at least a minute) with a sensitivity and specificity of 88% [95% confidence intervals 85, 90%] and 87% [85, 90%] 15 min before a hypotensive event. The area under the receiver operating characteristics curve was 0.95 [0.94, 0.95] [9]. The HPI algorithm better predicted hypotension than commonly used hemodynamic parameters trends including mean arterial pressure, stroke volume, and cardiac output [10]. Development and testing of this algorithm was based on invasive arterial line waveform data. However, only a small fraction of patients having noncardiac surgery require invasive arterial monitoring.

Advances in non-invasive hemodynamic monitoring now allow arterial pressure waveforms to be estimated from a finger cuff [11–16]. But whether the hypotension prediction algorithm, which incorporates subtle aspects of the arterial waveform, performs well from a non-invasive waveform estimate remains unknown. We therefore evaluated the performance of the Hypotension Prediction Index derived from non-invasive arterial pressure waveforms in non-cardiac surgical patients. We used ClearSight (formerly known as Nexfin) non-invasive finger blood pressure system in our investigation which gives reliable estimates of arterial pressure even in highly dynamic situations such as during induction of anesthesia [11] and clamping a carotid artery [12]. Reported difference between ClearSight and the invasive radial arterial pressure are small (2.2 ± 6.4 mmHg [11], and 3.5 ± 5.2 mmHg [12], for MAP), which is less than the 5 ± 8 mmHg criterion proposed by Association for the Advancement of Medical Instrumentation.

## Materials and methods

For this post hoc analysis, we used hemodynamic data measured by noninvasive finger cuff monitors (ClearSight, Edwards Lifesciences, Irvine, CA) in patients enrolled in a randomized trial of continuous noninvasive blood pressure monitoring during noncardiac surgery. The trial was approved by the Cleveland Clinic Institutional Review Board (IRB # 16-845) and written informed consent was obtained from all subjects participating in the trial. The trial was registered before patient enrollment at clinicaltrials.gov (NCT02872896, Principal investigator: Kamal Maheshwari, Date of registration: August 19, 2016) [17]. We enrolled 320 adults aged 45 years or older designated ASA physical status 3 or 4 who had moderate-to-high-risk non-cardiac surgery with general anesthesia between August 2016 and August 2017.

Patients were excluded if the attending anesthesiologist determined that invasive arterial monitoring was needed. Patients were also excluded when there was more than a 10% discrepancy in preoperative MAP between the arms, or if the expected duration of surgery exceeded 2 h.

The detailed trial protocol has been published [17]. Briefly, just before surgery patients were randomly allocated to continuous unblinded or blinded continuous blood pressure monitoring by an investigator not involved in clinical care, in a 1:1 ratio, using a reproducible set of computer-generated random number via a web-based system (REDCap secure web application). Allocation to blinded or unblinded continuous blood pressure monitoring was thus concealed as long as practical, and patients were not informed of their group assignments.

The continuous blood pressure monitor was properly positioned and all patients also had a cuff on opposite arms for intermittent oscillometric measurements. In the unblinded group, information from the continuous monitor was available to clinicians in addition to the usual oscillometric values. In the blinded group, blood pressure management was based only on intermittent oscillometric blood pressure monitoring per routine; information from continuous monitors was not available to the clinicians but recorded for analysis purposes. Oscillometric measurements were typically obtained at 5-min intervals, but clinicians were free to select any interval and to change it as conditions warranted. Clinicians were asked to minimize the amount and severity of hypotension < 65 mmHg MAP. However, the study protocol did not specify any particular approach. Clinicians were thus free to use any type and amount of intravenous fluids, whatever dose of vasopressors and inotropes they cared to and to adjust the inhalational concentration and intravenous anesthetic drugs as necessary.

The Hypotension Prediction Index was not available to either group and was calculated post hoc. But hypotension management and thus algorithm performance metrics might be influenced by the availability of continuous arterial pressure and the early treatment in the unblinded group. We therefore separately evaluated HPI performance in unblinded and blinded patients, as well as in the entire cohort.

### Statistical methods

The Hypotension Prediction Index is based on an algorithm that estimates the likelihood of a hypotensive event in the near future. The HPI algorithm takes arterial pressure waveform as the input, then extracts various waveform features, then together with the patient demographics (age, gender, height, and weight), it computes an index value that ranges between 0 and 100: the larger the value, the more likely and the sooner a hypotensive event will occur. For this study, raw arterial waveform data from our previous study [17], together with the patient demographics (age, sex, height, and weight) were passed into the HPI algorithm [9] to compute an HPI value every 20 s.

#### Predictive performance by receiver operating characteristics

Positives and negatives need to be defined for a receiver operating characteristic (ROC) analysis. We defined positive samples as data points exactly ‘t’ minutes (t = 5, 10, or 15) prior to a hypotension event, where a hypotensive event is defined as MAP ≤ 65 mmHg for at least 1 min. A negative sample was selected from each non-event segment of 30-min duration, where a non-event segment was at least 20 min from any hypotensive events and had MAP > 75 mmHg. The HPI model used in this noninvasive analysis was identical to the invasive HPI model developed previously [9]. Sensitivity and specificity (at an optimal threshold with the minimum difference between sensitivity and specificity) along with the ROC AUC were calculated from the positive and negative samples in the combined dataset. See Hatib et al. [9] for more details on this method of analysis for hypotension prediction.

#### Event rate analysis

In this analysis, we compared actual occurrences of hypotensive events to the predicted rate in 15 min and separately in the blinded, unblinded, and combined cohort. For every data point in a given HPI range, a 15-min forward search window was used to locate a hypotensive event. The percentage of all data samples with a hypotensive event compared to the total number of samples in the HPI bin was the rate of hypotension for that bin.

#### Positive predictive value

**Fig. 1:**
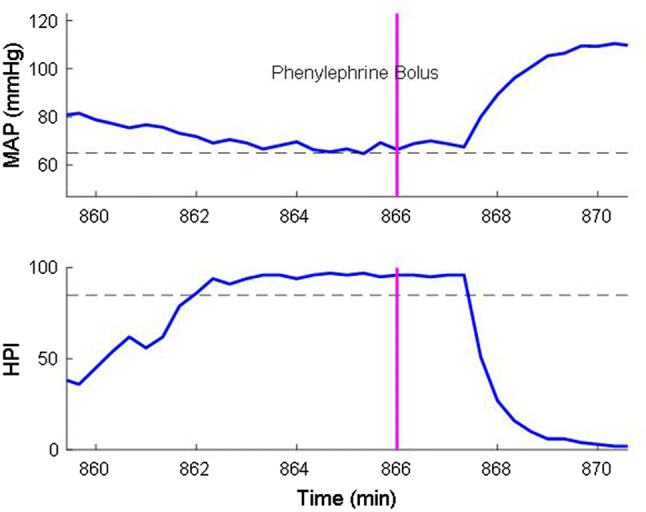
Effect of clinical intervention on HPI and MAP. *HPI* Hypotension Prediction Index, *MAP* mean arterial pressure

Positive predictive value of dynamic algorithms like HPI provides incomplete information because PPV does not take into account the continuous nature of monitoring [18]. A challenge is that clinical interventions in response to hypotension at mean arterial pressures exceeding 65 mmHg may result in false positive predictions. For example, in a 10 min window when HPI remains over 85 while the MAP is slowly decreasing, a clinical intervention might intervene to prevent MAP from decreasing to < 65 mmHg. See Fig. 1 for an example in which a bolus of phenylephrine, given after HPI reached 85, prevented a possible hypotension event. In this scenario, HPI might poossibly have correctly predicted hypotension save the vasopressor bolus, and the event should not be considered false-positive.

We tried to mitigate false-positive events by identifying rapid increases in MAP that were potentially consequent to clinical interventions. Specifically, we defined rapid mean pressure increases as > 5 mmHg in 20 s or > 8 mmHg in 2 min from a baseline MAP < 75 mmHg based on Hatib et al. [9] analysis. To determine the extent to which rapid blood pressure increases resulted from clinical interventions, we reviewed electronic records for surgical incision, and boluses of ephedrine, phenylephrine, or epinephrine within the preceding 5 min. Surgical manipulation, changes in anesthesia level, fluid bolus, etc. can also cause a rapid increase in blood pressure, however, we could not reliably confirm these interventions from EMR data. Our mitigation strategy was only used to estimate positive predictive value. Because receiver operating characteristics analyses are based on the sensitivity (true-positives) and specificity (true negatives), no assumptions were required.

We calculated the positive predictive value (PPV) at an HPI threshold of 85. We selected an HPI threshold of 85 since the current commercial implementation includes an alarm at this value. True positives were all data samples with HPI value above 85 and a hypotensive event within 15 min in the future. False positives were all data samples with HPI value above 85 but without a hypotensive event within 15 min in the future. PPV was calculated as the ratio of true positives to all data points with HPI value above 85. We assumed rapid increases in blood pressure were true positives only when there was supportive evidence in the medical record, and eliminated other blood pressure increases from the analysis.

All statistics were performed with MATLAB (version R2018a; The Mathworks Inc, Natick, MA). Repeated measurents from same subjects were evlauated  by bootstrapping method [19], where a total number of all subjects were first randomly chosen from all subjects with replacements, then statistics were computed. This process was repeated 2000 times from which the standard error was calculated. The bootstrap confidence interval was calculated as a 95% asymptotic confidence interval.

## Results

Four patients enrolled in the underlying trial did not receive the allocated intervention and 11 others had incomplete waveform data. Consequently, a total of 305 of patients were included in this analysis, with an average age 60 ± 9 years, weight 98 ± 28 kg and height 171 ± 10 cm. The incidence and severity of hypotension are characterized by various way in Table 1. Patients averaged 3.3 clinical interventions for hypotension (skin incision or vasopressor administration). Among patients in whom mean pressure increased > 5 mmHg in 20 s or > 8 mmHg in 2 min from a baseline MAP < 75 mmHg, 46% had skin incision or were give a vasopressor bolus within 5 min.

**Table 1:** Hypotension statistics in all patients as median [25th, 75th] percentiles

Summary statistics	Combined	Blinded	Unblinded
Monitoring time (min)	202 [154, 258]	202 [154, 246]	202 [157, 266]
Number of patients with hypotension (event)	187 of 305 (61%)	94 of 150 (62%)	93 of 155 (60%)
Total number of events in dataset	649	337	312
Average number of events per patient	1 [0, 3]	1 [0, 4]	1 [0, 3]
Average duration of each event (min)	2 [1, 5]	3 [1, 5]	2 [1, 4]
Total duration of events per patient (min)	2.3 [0, 9.4]	3.5 [0, 12.3]	1.7 [0, 7.3]
Total duration of events per patient (%)	1.1 [0, 5.0]	1.6 [0, 7.4]	0.8 [0, 3.5]
Mean MAP under 65 mmHg per patient (mmHg)	60 [58, 62]	60 [57, 62]	61 [58, 62]
Area under 65 mmHg for MAP per patient (mmHg × min)	11.9 [0, 64.4]	18.0 [0, 79.1]	9.3 [0, 49.3]
Time weighted area (MAP < 65 mmHg) per patient (mmHg)	0.05 [0, 0.35]	0.08 [0, 0.53]	0.04 [0, 0.24]

### Algorithm performance

**Fig. 2:**
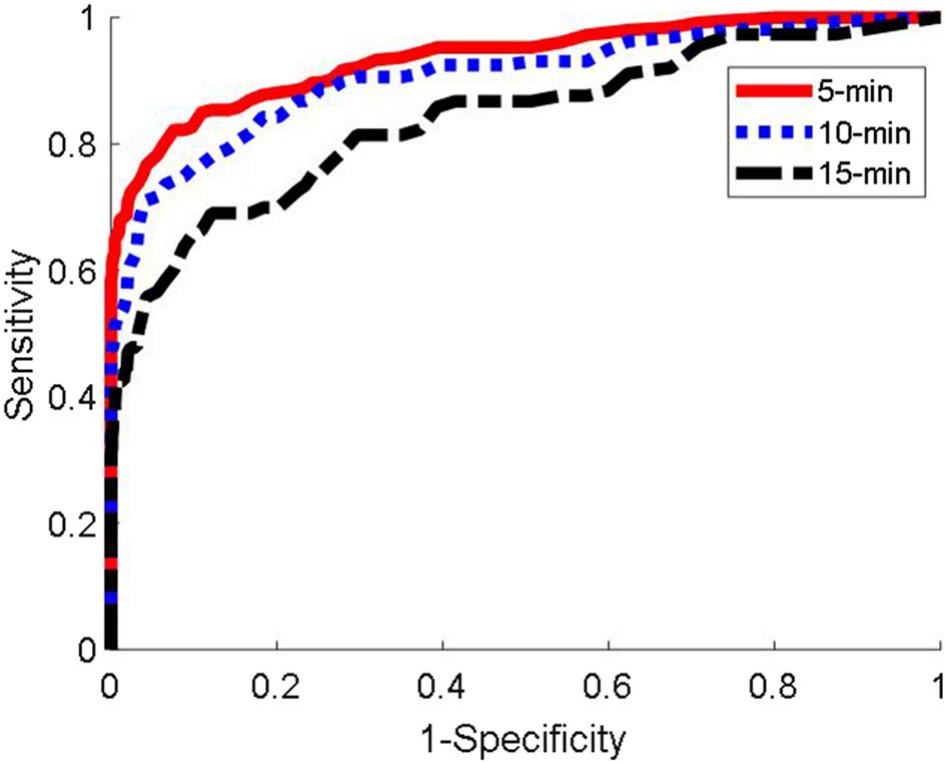
ROC plot at 5, 10, and 15-min for the entire cohort of 305 patients

Figure 2 shows the receiver operating characteristic curves for the 5-, 10-, and 15-min prediction time points before each hypotensive episode, defined by MAP < 65 mmHg for at least a minute, using the entire dataset (N = 305). Table 2 summarizes the ROC results (sensitivity, specificity, and AUC) separately for patients assigned to blinded and unblinded continuous pressure monitoring.

**Table 2:** Receiver operating characteristics area under the curve analysis in all patients at 5, 10, and 15 min before a hypotensive event defined by mean arterial pressure (MAP) < 65 mmHg sustained for a minute

Time	AUC [95% Conf]	Sensitivity	Specificity	Threshold
Combined (N = 305)				
5 min	0.93 [0.91,0.95]	0.86 [0.82,0.89]	0.86 [0.82,0.89]	30
10 min	0.90 [0.87,0.93]	0.83 [0.79,0.86]	0.83 [0.79,0.86]	28
15 min	0.84 [0.79,0.88]	0.75 [0.71,0.80]	0.75 [0.71,0.80]	22
Blinded arm (N = 150)				
5 min	0.94 [0.90,0.96]	0.86 [0.82,0.91]	0.87 [0.82,0.91]	30
10 min	0.93 [0.88,0.96]	0.87 [0.81,0.92]	0.87 [0.81,0.91]	28
15 min	0.83 [0.75,0.89]	0.75 [0.68,0.82]	0.75 [0.68,0.81]	22
Unblinded arm (N = 155)				
5 min	0.93 [0.91,0.95]	0.86 [0.82,0.89]	0.86 [0.82,0.89]	31
10 min	0.90 [0.87,0.93]	0.83 [0.79,0.86]	0.83 [0.79,0.86]	31
15 min	0.84 [0.79,0.88]	0.75 [0.71,0.80]	0.75 [0.71,0.80]	22

Sensitivity and specificity are given at the optimal threshold for HPI

**Fig. 3:**
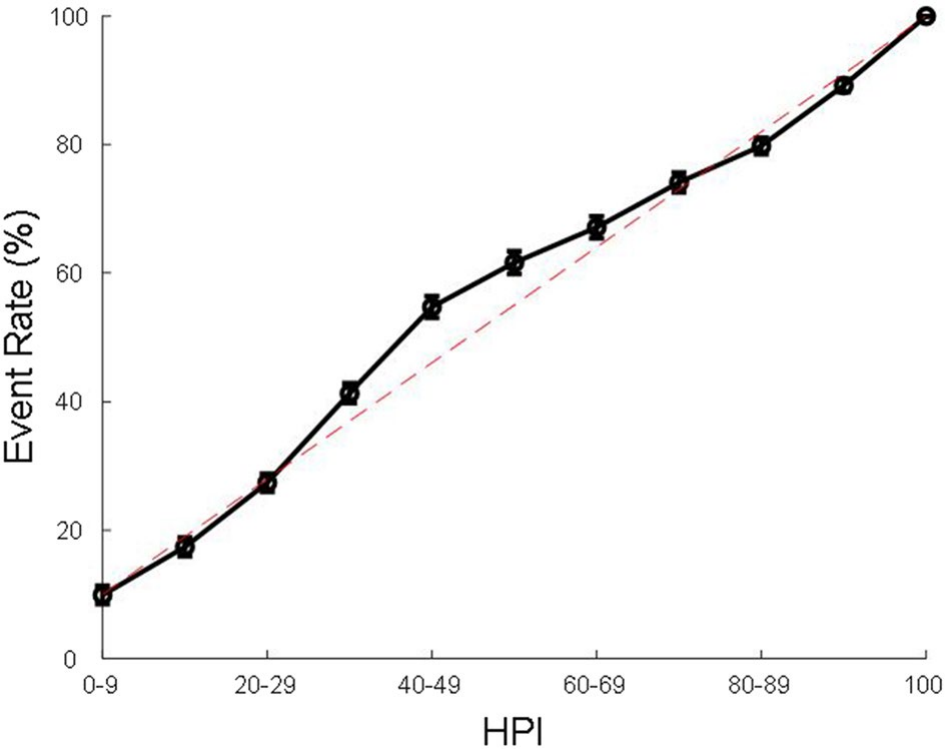
Hypotensive, MAP < 65 mmHg, event rates in all patients. Dashed lines are the lines of identity. *MAP* mean arterial pressure

Figure 3 shows occurrence of hypotension against HPI for all patients. The amount of hypotension increases linearly with the increase in the algorithm prediction index. The results along with the median time to hypotension and 25th, 75th percentiles as an indication for range, for different ranges of HPI is shown in Table 3 and Supplement Fig. 1 for the entire dataset, and the blinded and unblinded arms separately. A Hypotension Prediction Index of 80–89 provided a median of 6.0 [95% confidence interval 5.3, 6.7] minutes warning before mean arterial pressure decreased to < 65 mmHg.

**Table 3:** Event rate analysis

HPI range	Event rate (95% CI), %	Median time to event (95% CI), min	25th percentile time to event (95% CI), min	75th percentile time to event (95% CI), min	Number of samples
Combined (N = 305)					
0–9	9.9 [8.6, 11.3]	9.7 [8.7, 10.3]	6.3 [5.7, 7.0]	12.3 [12.0, 13.0]	5715/57,983
10–19	17.5 [15.4, 19.7]	9.0 [8.3, 10.0]	6.0 [5.3, 6.7]	12.7 [12.0, 13.0]	5689/32,581
20–29	27.4 [24.8, 30.3]	8.7 [8.0, 9.0]	5.3 [4.7, 6.0]	11.7 [11.3, 12.3]	6882/25,082
30–39	41.3 [38.0, 44.8]	8.3 [8.0, 8.7]	5.3 [5.0, 5.7]	11.3 [11.0, 12.0]	9635/23,286
40–49	54.7 [50.9, 58.4]	7.4 [7.0, 8.1]	4.7 [4.0, 5.0]	10.7 [10.0, 11.0]	5562/10,182
50–59	61.7 [57.6, 65.5]	7.3 [6.7, 7.7]	4.3 [4.0, 5.0]	11.0 [10.0, 11.7]	3843/6239
60–69	67.1 [62.9, 71.1]	6.7 [6.0, 7.7]	3.7 [3.3, 4.1]	11.0 [10.0, 12.0]	3601/5382
70–79	74.1 [70.0, 78.1]	6.7 [5.7, 7.3]	3.7 [3.3, 4.3]	10.3 [9.7, 11.0]	3396/4598
80–89	79.9 [75.9, 83.6]	6.0 [5.3, 6.7]	3.3 [3.0, 3.7]	9.7 [9.0, 10.3]	4091/5127
90–99	89.2 [86.3, 92.1]	2.7 [2.3, 3.0]	1.0 [1.0, 1.0]	6.7 [6.0, 7.3]	14,007/15,726
100	100 [100, 100]	0 [0, 0]	0 [0, 0]	0 [0, 0]	5690/5728
Blinded arm (N = 150)					
0–9	9.9 [8.2, 11.9]	9.7 [8.3, 10.7]	6.3 [6.0, 7.0]	12.3 [11.7, 13.0]	2899/29,445
10–19	16.5 [13.5, 20.0]	9 [7.7, 10.1]	5.7 [5.0, 6.7]	12.7 [11.3, 13.3]	2616/15,875
20–29	25.1 [21.8, 28.8]	8.7 [7.7, 9.3]	5.3 [4.3, 6.1]	12.0 [11.3, 12.3]	2929/11,651
30–39	40.0 [35.2, 44.7]	8.3 [7.7, 9.0]	5.3 [5.0, 5.7]	11.3 [10.7, 12.0]	4540/11,392
40–49	54.0 [48.2, 59.6]	7.3 [6.7, 8.3]	4.3 [4.0, 5.0]	10.3 [9.8, 11.3]	2490/4625
50–59	63.8 [58.6, 69.3]	7.7 [6.7, 8.7]	4.3 [3.7, 5.3]	11.3 [10.0, 12.0]	1749/2742
60–69	68.7 [63.9, 74.1]	7.7 [6.3, 8.3]	3.7 [3.3, 4.7]	11.3 [10.3, 12.3]	1623/2357
70–79	74.6 [69.3, 80.2]	6.7 [6.0, 8.0]	3.7 [3.0, 4.7]	10.7 [9.3, 11.3]	1578/2116
80–89	79.7 [73.0, 85.9]	6.3 [5.3, 7.3]	3.3 [3.0, 4.0]	10.0 [8.8, 11.0]	1842/2317
90–99	90.2 [86.3, 93.8]	2.7 [2.3, 3.0]	1 [1.0, 1.3]	6.7 [6.0, 7.7]	6786/7536
100	100 [100, 100]	0 [0, 0]	0 [0, 0]	0 [0, 0]	3292/3292
Unblinded arm (N = 155)					
0–9	9.9 [8.1, 11.9]	9.3 [8.0, 10.7]	6.0 [4.8, 7.3]	12.3 [11.7, 13.3]	2828/28,512
10–19	18.5 [15.6, 21.4]	9.0 [8.0, 10.7]	6.0 [5.3, 7.3]	12.3 [11.7, 13.0]	3073/16,696
20–29	29.6 [25.2, 33.9]	8.3 [7.7, 9.5]	5.7 [4.7, 6.3]	11.7 [10.7, 12.3]	3947/13,404
30–39	43.1 [38.4, 47.5]	8.3 [7.7, 9.0]	5.2 [4.3, 5.7]	11.6 [11.0, 12.3]	5068/11,819
40–49	55.5 [50.7, 60.3]	7.7 [6.7, 8.7]	4.7 [4.0, 5.3]	10.7 [10.0, 11.3]	3064/5524
50–59	60.2 [53.9, 66.0]	7.0 [6.0, 7.8]	4.3 [4.1, 5.0]	10.7 [9.6, 11.7]	2087/3466
60–69	66.0 [59.7, 72.4]	6.0 [5.3, 7.3]	3.3 [3.0, 4.3]	10.3 [8.7, 12.0]	1974/2991
70–79	73.9 [68.4, 79.5]	6.5 [5.3, 7.7]	3.7 [3.0, 4.0]	10.0 [9.0, 11.0]	1815/2459
80–89	80.1 [75.3, 85.3]	6.0 [5.0, 6.7]	3.3 [2.7, 4.0]	9.3 [8.0, 10.3]	2238/2794
90–99	88.5 [83.9, 92.6]	2.7 [2.3, 3.0]	1.0 [1.0, 1.0]	6.3 [5.0, 7.3]	7232/8179
100	100 [100, 100]	0 [0, 0]	0 [0, 0]	0 [0, 0]	2425/2425

*HPI* Hypotension Prediction Index

The positive predictive values of the algorithm were similar in the blinded and unblinded groups. We thus present results for the entire population at an HPI threshold of 85. The positive predictive value prediction was 0.83 (95% confidence interval [0.79, 0.87]) when rapid increases in blood pressure after HPI reached 85 were considered to be true positives only when there were supportive clinical interventions in the electronic record, and other rapid increases, presumably due to surgical manipulations, fluid boluses, change in anesthetic level, etc. were excluded from the analysis altogether..

## Discussion

Our primary result is that the Hypotension Prediction Index, which was developed and validated from invasive arterial waveforms, works reasonably well with non-invasive arterial pressure waveform estimates. Given that the vast majority of noncardiac surgical patients are monitored non-invasively, our findings markedly broaden the range of patients who might benefit.

The non-invasive ClearSight system used in our investigation gives reliable estimates of arterial pressure even in highly dynamic situations such as during induction of anesthesia [11] and clamping a carotid artery [12]; the reported difference between invasive radial arterial pressure was small (2.2 ± 6.4 mmHg [11], and 3.5 ± 5.2 mmHg [12], for MAP), which is below the 5 ± 8 mmHg of AAMI acceptable criteria for pressure measurements. While the blood pressures obtained noninvasively from the non-invasive system are relatively accurate and reliable, the sensitivity and specificity of HPI prediction was slightly lower compared to invasive arterial pressure. The lower sensitivity and specificity presumably result from subtle differences in the waveform which are used to calculate HPI [9]. The features used in the HPI model come from detailed waveform features within a heart-beat, variability of features over short periods of time [9], and critically depend on a good intra-patient precision. The non-invasive system has good intra-patient precision in tracking subtle changes in waveforms and pressure levels [11, 12]. Perhaps consequently, HPI performed well on the noninvasive waveforms suggesting noninvasive waveform features are close to those of invasive waveforms. The quality of noninvasive waveforms can be influenced by finger perfusion and extreme hemodynamic conditions. Algorithm retraining using a larger dataset of noninvasive waveforms from a wider patient population may further improve its accuracy.

Positive predictive value of dynamic algorithms like HPI provides incomplete information because PPV does not take into account the continuous nature of monitoring and possible interventions [18]. Clinical intervention influencing blood pressure, drugs or surgical manipulation, can cause abrupt changes in blood pressure resulting in false positive predictions. Moreover, the only way to evaluate HPI without the clinician's intervention would be a blind prospective evaluation, and to force the clinician to let the MAP go down until 65 mmHg or below, without any intervention, which is unethical. Nonetheless, the PPV was 0.83 [0.79, 0.87] when rapid increases in blood pressure were considered to be true positives only when supportive clinical interventions in the electronic record were present, and other rapid increases, presumably due to surgical manipulations, fluid boluses, change in anesthetic level, etc. were excluded from the analysis altogether. Without assumptions, then, four-fifth of high HPI episodes resulted in either hypotension or a clinical intervention to prevent hypotension.

Clinicians use a wide variety of hemodynamic measurements and experience to anticipate intraoperative blood pressure changes. Factors related to surgical procedures, anesthetic drugs, and advanced hemodynamic parameters when available all guide clinicians to manage blood pressure. Stroke volume variation [20–22], pulse pressure variation [23, 24], and systolic pressure variations [25–28] are often used to assess fluid responsiveness and guide fluid administration. Stroke volume, dP/dt_max_ [29, 30], and cardiac output all reflect cardiac function, systemic vascular resistance, and dynamic arterial elastance [31]. All help manage hypotension, but none of them reliably predicts hypotension. The Hypotension Prediction Index uniquely predicts a future hypotensive state, and on average provides 6 min of warning which will often be sufficient to administer a vasopressor. If a clinician wants a longer predictive time, a lower threshold for HPI may be used, which will extend the time available to determine the proper pro-active treatment to avoid hypertension. The extent to which hypotension prediction reduces intraoperative hypotension and potentially reduces serious complications needs to be formally evaluated. Trial is already in progress (NCT03610165).

In summary, the Hypotension Prediction Index, which was developed and validated with invasive arterial waveforms, predicts intraoperative hypotension reasonably well from non-invasively measured arterial blood pressure waveforms. Being able to use non-invasive pressure waveforms will widen the range of patients who might benefit.

## Electronic supplementary material

Below is the link to the electronic supplementary material.

Electronic supplementary material 1 (JPG 51 kb) HPI behavior prior to a hypotensive event; A. All events, thick line is the mean, B. Mean and standard deviation. HPI, hypotension prediction index
