# Supplementary figures and images for: Performance of the Hypotension Prediction Index with non-invasive arterial pressure waveforms in non-cardiac surgical patients
Source: J Clin Monit Comput. 2020 Jan 27;35(1):71–8. doi: 10.1007/s10877-020-00463-5 (PMC7889685; doi:10.1007/s10877-020-00463-5)

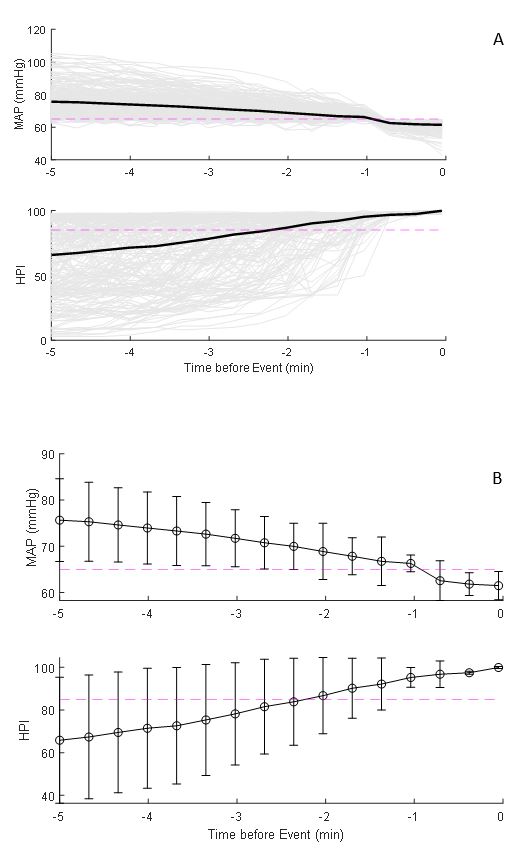

Supplement: Supplementary file 1 — Electronic supplementary material 1 (JPG 51 kb) HPI behavior prior to a hypotensive event; A. All events, thick line is the mean, B. Mean and standard deviation. HPI, hypotension prediction index [file 10877_2020_463_MOESM1_ESM.jpg]
